# Design and validation of a questionnaire on the perception of cheating in traditional sporting games: CHEAT-1

**DOI:** 10.3389/fpsyg.2025.1661933

**Published:** 2025-09-26

**Authors:** Unai Sáez de Ocáriz, Pere Lavega-Burgués, Miguel Pic

**Affiliations:** ^1^Motor Action Research Group (GIAM), National Institute of Physical Education of Catalonia (INEFC), University of Barcelona (UB), Barcelona, Spain; ^2^Motor Action Research Group (GIAM), National Institute of Physical Education of Catalonia (INEFC), University of Lleida (UdL), Lleida, Spain; ^3^Motor Action Research Group (GIAM), University of Valladolid, Soria, Spain

**Keywords:** motor conduct, fair play, values education, student perceptions, moral reasoning, educational assessment

## Abstract

**Introduction:**

Cheating in traditional sporting games (TSG) presents a significant challenge for values education and harmonious school coexistence. Despite its educational relevance, no validated instruments are currently available to assess students’ perceptions of cheating in TSG contexts.

**Methods:**

This study aimed to design and validate the CHEAT-1 questionnaire, designed to assess perceptions of cheating in TSG. The instrument was created through a four-stage process involving item construction based on the internal and external logic of motor games, expert panel reviews (*n* = 13), and focus groups with students from primary, secondary, and university levels (*n* = 24). The preliminary version (46 items) was reduced to a final version of 18 items, structured in two dimensions: Internal Logic and External Logic. A sample of 564 students aged 10–30 completed the questionnaire.

**Results:**

Exploratory and confirmatory factor analyses supported a two-factor structure comprising Internal Logic and External Logic dimensions. The model demonstrated strong fit indices (RMSEA = 0.05; CFI = 0.98; TLI = 0.97). Internal consistency was high, with Cronbach’s alpha and McDonald’s omega coefficients both reaching 0.96. Content validity was confirmed, with all items exceeding a CVI of 0.80.

**Discussion:**

The CHEAT-1 instrument demonstrates strong psychometric properties and fills a critical gap in the assessment of ethical behavior in Physical Education settings. Its application can support teachers in detecting students’ perceptions of cheating and implementing targeted pedagogical interventions. The tool offers a valuable resource for future research and practice in values-based education across different educational stages.

## 1 Introduction

This study introduces a questionnaire designed for teachers of Physical Education, Physical Activity, and Sport, with the aim of assessing the perception of cheating in motor games. The tool is conceived to support educational development and foster school coexistence, both of which are fundamental in addressing current societal challenges. Within this framework, educational institutions are recognized as key environments for fostering learning in peace, inclusion, and democracy. According to UNESCO (2014), Physical Education, Physical Activity, and Sport promote respectful and participatory communities through formative motor practices oriented toward ethical and social development.

School coexistence, recognized as an educational pillar in the Delors Report through the principle of “learning to live together” (Delors, 1996), nurtures social competence and peaceful conflict resolution (Durlak et al., 2011; López-Castedo et al., 2018). Physical Education can play a significant role in cultivating positive school environments through motor experiences that promote respect, active participation, and the development of healthy relationships (Del Rey et al., 2009; Domitrovich et al., 2017; Kirk, 2020; Menéndez Santurio and Fernández-Río, 2016).

Ongoing cultural, social, and educational transformations highlight the need for schools to go beyond academic competencies and foster social skills, emotional intelligence, and relational competence—key attributes for shaping empathetic and socially engaged citizens. These dimensions must be addressed from early education, through intentional and developmentally appropriate pedagogical strategies. Learning environments based on cooperation, mutual respect, and equity lay the groundwork for a holistic education aligned with the challenges of the 21st century (Cejudo et al., 2020; Greenberg et al., 2017; Oberle et al., 2016; Bücker et al., 2018; Panayiotou et al., 2019; Teraoka et al., 2021).

In this regard, ethical behavior in Physical Education, Physical Activity, and Sport has gained relevance due to its association with fair play, rule violations, and antisocial conducts. These contexts can serve as fertile ground for developing prosocial attitudes or, conversely, for encouraging behaviors that undermine shared norms (Latorre-Román et al., 2020). According to Kavussanu and Boardley (2009), “sport and physical activity provide unique opportunities for young people to either develop or erode their sense of morality.” Understanding how students perceive cheating in motor games is therefore essential for promoting fairer, more respectful practices in educational contexts.

### 1.1 School coexistence, physical education, and motor games

Motor games represent a fundamental expression of human action within educational, recreational, and social contexts. Due to their playful nature and internal logic, they serve as a powerful pedagogical tool in the fields of Physical Education, Physical Activity, and Sport (Parlebas, 2001). These games contribute to the students’ development - physically, cognitively, emotionally, and socially-, while facilitating meaningful learning and the transmission of values through interactions with the environment and others (Latorre-Román et al., 2020).

Values such as coexistence, respect, equity, and peace can be meaningfully developed through well-structured motor experiences. However, these experiences may be disrupted by conducts that violate the internal logic of the game—such as cheating—thus undermining trust and compromising group fairness (Gibbons et al., 1995). The school environment, particularly during the critical developmental period between ages of 11 and 16, provides a strategic setting for promoting such values and for fostering meaningful learning, that positively impacts students’ coexistence, well-being, and social engagement (Eisman et al., 2016; Hromek and Roffey, 2009; Martínez Sánchez et al., 2019; Norwalk et al., 2016; Herrera et al., 2016).

Like any other social space, schools are not exempt from interpersonal conflicts, which can affect the school climate and the quality of peer relationships (Frías-Armenta et al., 2018). Rather than being avoided, these conflicts can be transformed into pedagogical opportunities to strengthen interpersonal relationships. When addressed from an educational perspective, they foster the development of key social competencies needed for constructive coexistence (Lederach, 1995).

In this regard, physical activity and sport within the school setting offer effective strategies for promoting positive peer interactions (Bukowski et al., 2007). These experiences enhance not only communicative and prosocial skills, but also mutual respect, recognition of others, and cooperation among participants (Trigueros et al., 2019). Thus, motor practice becomes a meaningful pathway for students’ holistic development.

Through engagement in motor experiences, students develop cognitive, emotional, and social capacities, facilitating the construction and reflection of values and moral judgment in relation to game rules and their consequences (Bermejo et al., 2019; Hodge and Lonsdale, 2011). UNESCO has highlighted the educational potential of Physical Education as a subject that integrates motor learning with values education (UNESCO, 2015, 2013).

Physical Education is grounded in procedural experiences where students learn by doing. This learning involves mastering motor conducts consistent with the internal logic of each game, which requires interaction with space, time, objects, and others (Parlebas, 2001; Lagardera and Lavega, 2005). These conducts are not merely physical responses but also expressions of ethical and relational attitudes, making motor games a privileged context for values education and the promotion of positive school coexistence.

### 1.2 Cheating as a deviant motor conduct

Motor conduct, understood as bodily actions with communicative or functional intent, involves both technical-tactical decisions and ethical stances toward rules and others (Martinek and Lee, 2012). In this context, cheating can be influenced by both situational and personal factors. Although aspects such as prosocial behavior, moral disengagement, and fair play have been studied (Kavussanu and Boardley, 2009; Boardley and Kavussanu, 2007; Nuñez et al., 2006), there is currently no validated instrument specifically designed to assess the perception of cheating in motor games.

The concept of motor conducts recognizes that each bodily action engages students’ personality across physical, emotional, cognitive, and social dimensions. Conceiving Physical Education as the Pedagogy of motor conduct enables the design of holistic experiences that foster students’ competency development (Sáez de Ocáriz et al., 2013). In this regard, traditional sporting games (TSG) serve as valuable pedagogical resources to transform conflictive conducts into educational opportunities and to promote values through relational well-being (Sáez de Ocáriz, 2011).

In TSG, students face situations that must be resolved according to the internal logic of the game, generating a wide range of relational challenges and making these experiences powerful spaces for meaningful social interaction (Parlebas, 2001). When motor conducts align with the rules, coexistence is enhanced; however, in situations of tension, behaviors that hinder coexistence may arise, thereby highlighting the need for intentional pedagogical intervention (de Sáez Ocáriz and Lavega, 2013).

Understanding cheating in motor games involves analyzing both the transgression of rules and the motivations behind such conduct. These actions, by breaking the internal logic of the TSG, can generate interpersonal conflict and disrupt group dynamics. Their analysis can be approached from two complementary perspectives: internal logic, which refers to adherence to rules-based dimensions related to space, time, materials, and peers; and external logic, which involves the social motivations underlying compliance with or deviation from the rules (Parlebas, 2001).

The external logic of cheating in TSG can be interpreted through the lens of several motivational theories. Self-Determination Theory distinguishes between extrinsic motivations (e.g., seeking external recognition) and intrinsic motivations (e.g., striving for competence) (Deci and Ryan, 1985, 2002; Ryan and Deci, 2000). This theory identifies three basic psychological needs required to achieve optimal well-being and development: (i) Autonomy, understood as the individual’s need to feel like the author of their own behavior, (ii) Competence, for the need to be effective in interactions with the environment, demonstrating skills and task mastery, and finally (iii) Relatedness, for feeling connected or bonded with other people. Social identity theory suggests that actions are aimed at maintaining group status (Tajfel, 1982; Tajfel and Turner, 1979). This theory explains how group membership influences self-concept and behavior. Social Comparison Theory interprets behavior as a means of seeking validation (Festinger, 1954; Suls et al., 2002), since people have an innate need to evaluate themselves by comparing themselves with others. Finally, Achievement Goal Theory associates cheating with the pursuit of performance outcomes and visible success (Dweck and Leggett, 1988; Elliot and Dweck, 2005). The theory distinguishes between a performance orientation, which focuses on comparison with others, and a task orientation (or mastery) which emphasizes personal improvement and skill mastery.

Currently, the lack of specific instruments to analyze the perception of cheating in motor games represents a significant gap in the scientific literature. Although there are scales that address ethical dimensions such as prosocial behavior or sports morality (Gutiérrez-Marín et al., 2017; Kavussanu, 2006; Shields et al., 1995), none specifically focus on cheating as a form of motor conduct. This limitation hinders the advancement of knowledge and reduces teachers’ ability to intervene in a well-founded manner in the ethical development of students through motor activities in educational contexts.

Considering the identified theoretical and methodological gap, the present study aims to design and validate the CHEAT-1 questionnaire for assessing students’ perceptions of cheating in traditional sporting games. The development process is grounded in a solid theoretical framework and integrates expert judgment, student feedback, and advanced psychometric analyses to ensure content validity, structural coherence, and internal reliability. The resulting instrument is intended to serve both researchers and educators, providing a scientifically sound tool for diagnosing students’ attitudes and beliefs regarding ethical behavior and fair play in educational motor contexts.

## 2 Materials and methods

The methodological process was structured into three main phases: (1) development and refinement of questionnaire items; (2) data collection and psychometric validation; and (3) analysis of reliability and internal structure. Each phase followed internationally recognized standards for scale development and validation (American Educational Research Association, American Psychological Association, and National Council on Measurement in Education, 2014; Boateng et al., 2018) and incorporated both theoretical foundations and empirical procedures to ensure the instrument’s validity and reliability.

### 2.1 Phase 1. Instrument development

The objective of this phase was to develop an initial pool of items for the CHEAT-1 questionnaire and to gather evidence related to its content validity and validity based on response processes (American Educational Research Association, American Psychological Association, and National Council on Measurement in Education, 2014).

Following the recommendations of Boateng et al. (2018), combined strategies were used to develop and select the most appropriate items, integrating deductive approaches (theoretical review) and inductive approaches (researcher discussions). Efforts were made to ensure that the items accurately reflected the domain of interest through expert panels, and that they were understandable to the target population through focus groups with university students, secondary school students, and upper primary students.

#### 2.1.1 Participants

A total of 13 researchers participated in two expert panels (9 men and 4 women), with a mean age of 40.8 years (*SD* = 10.8; *range* = 26–61 years). The first panel included 8 experts with diverse profiles: a university full professor, an associate professor, a temporary lecturer, and a predoctoral student—all specialized in motor games—as well as a secondary and high school teacher and a primary school teacher, both with a specialization in Physical Education.

The second panel consisted of five external experts with teaching and research experience in the university setting: two associate professors specialized in the topic, two experts in research methodology, and one specialist in formal education.

Subsequently, 24 students participated across three focus groups. The university group (first-year students in the Bachelor’s Degree in Physical Activity and Sport Sciences) included 4 males and 4 females (*mean age* = 22.63 years; *SD* = 2.07; *range* = 7 years). The compulsory secondary education (ESO) group included students from all four grade levels (1 male and 1 female per grade), with a mean age of 14.04 years (*SD* = 1.5; *range* = 5 years). The primary education group (upper cycle) included 4 boys and 4 girls (*mean age* = 10.75 years; *SD* = 0.71; *range* = 2 years).

#### 2.1.2 Procedure

The study was conducted in accordance with the Declaration of Helsinki and was approved by the Bioethics Committee of the University of Barcelona (CER122415). All participants signed an informed consent form prior to their participation. The first phase included four stages, and the items developed were based on key concepts identified in the literature, from both the internal and external logic perspectives of motor games.

##### 2.1.2.1 Stage 1

An initial item pool for the questionnaire was developed based on the theoretical framework. The preliminary list included items related to the internal logic of motor games, the external logic, and the social motivation for cheating. Three criteria were applied: two theoretical (reference to the internal and external logic of motor games) and one linguistic (item comprehensibility). The items were written in Spanish and translated into English through a forward and backward translation process (back-translation) carried out by four researchers until the final version was agreed upon.

##### 2.1.2.2 Stage 2

Two expert panels were convened. The first panel used a discussion group technique to review and select the initial items, focusing on their wording, meaning, and potential redundancies. Twenty-one days in advance, the panel received documentation outlining the questionnaire’s objective, the theoretical framework, the design structure, and the proposed items. The group met in two sessions lasting 1.5 h each (3 h in total), and following the discussion, item selection for validation was agreed upon.

The second panel conducted an individual review, providing written feedback on the relevance, clarity, appropriateness, and importance of each item. They received the same documentation as the first panel, along with the validation protocol and evaluation form. They were given 40 days to submit their reports, and the documents were provided in various formats (Word, PDF, Excel).

Finally, the authors analyzed the feedback, reviewed the suggestions, and defined the final items for both instruments.

##### 2.1.2.3 Stage 3

Three focus groups were conducted to assess the comprehension of the selected items. Students were grouped by educational level (university, secondary, primary), with balanced gender representation. The sessions, lasting approximately 40 min, were held on different days. Participants were informed in advance about the study’s objectives and provided informed consent. Then, students reviewed the items and identified any they did not understand. A discussion followed to ensure item clarity. With authorization, the sessions were recorded for later analysis.

##### 2.1.2.4 Stage 4

A thorough review of the development process was carried out to ensure that all key recommendations provided by experts had been systematically addressed. This led to the construction of the preliminary version of the instrument, named CHEAT-1.

#### 2.1.3 Results and discussion

In response to the lack of specific instruments for assessing perceptions of cheating in TSG, the CHEAT-1 questionnaire was developed through a rigorous four-stage process, grounded in theoretical foundations and validated through expert input and empirical testing.

##### 2.1.3.1 Stage 1

The authors generated an initial pool of 68 potential items, grounded in key theoretical concepts related to the internal and external logic of TSG.

##### 2.1.3.2 Stage 2

A first panel of experts reviewed the 68 items generated in Stage 1 and proposed modifications regarding wording, conceptual appropriateness, and potential redundancy. As a result, 52 items were reformulated to enhance clarity and address comprehension issues, and 4 new items were added (*CVI* = 0.87).

A second panel of experts subsequently assessed the items in terms of relevance, clarity, appropriateness, and perceived importance. Following this evaluation, 34 items were modified and 10 were removes, concluding the stage with a total of 46 items (*CVI* = 0.89).

##### 2.1.3.3 Stage 3

Focus groups involving students from primary, secondary, and university levels were conducted to assess item comprehensibility. As a result of this analysis, 33 items were adjusted due to comprehension difficulties, using vocabulary appropriate and accessible for university, secondary, and primary students. Following this process, all 46 items were retained, deemed suitable for use in assessing the perception of cheating in TSG.

##### 2.1.3.4 Stage 4

The authors reviewed the entire process to ensure that all key suggestions from the previous phases were properly incorporated. It was determined that no further adjustments were necessary, and no additional items were added or removed.

The process concluded with a total of 46 items, formalizing the preliminary version of the CHEAT-1 questionnaire – Version 1.

### 2.2 Phase 2. Data collection and internal structure of the questionnaire

The objective of this phase was to explore and refine the structure of the preliminary version of the CHEAT-1 questionnaire – Version 1, and to provide evidence of its validity based on internal structure. Given that the questionnaire was designed as a bidimensional scale, a confirmatory factor analysis (CFA) approach was employed. In addition, its reliability and concurrent validity were assessed.

#### 2.2.1 Participants

The study included 564 students (53.59% male and 46.41% female), of whom 138 were from the final cycle of Primary Education (55.80% male and 44.20% female; *mean age* = 10.55; *SD* = 0.65; *age range* = 10–12 years), 189 from Compulsory Secondary Education (51.09% male and 48.91% female; *mean age* = 13.55; *SD* = 1.17; *age range* = 12–16 years), and 237 from the first year of university (54.40% male and 45.60% female; *mean age* = 19.03; *SD* = 1.78; *age range* = 18–30 years).

#### 2.2.2 Instruments

This questionnaire measures students’ perception of cheating in traditional sporting games. Participants completed Version 1 derived from Phase 1, which consists of 18 items organized into a bifactorial structure, using a five-point Likert scale with anchors ranging from 1 (strongly disagree) to 5 (strongly agree).

#### 2.2.3 Procedure

Informed consent was obtained from all participants; in the case of minors, schools and families were contacted to obtain such consent. Students were informed about the purpose of the study, encouraged to respond honestly, and assured of the confidentiality of their data. Both students and their families provided informed consent.

The estimated time to complete the questionnaire was 12 min. Although the researchers were not physically present during the sessions, the responsible teachers-maintained telephone contact with the research team to resolve any questions.

#### 2.2.4 Data analysis

First, reliability analyses were conducted using Cronbach’s alpha (Cronbach, 1951) and McDonald’s omega coefficients to assess the internal consistency of the questionnaire items. Subsequently, the suitability of the data for factor analysis was verified using the Kaiser-Meyer-Olkin (KMO) index and Bartlett’s test of sphericity, both of which showed significant values. Based on these results, an exploratory factor analysis was carried out to identify the underlying structure of the construct. Finally, a confirmatory factor analysis was conducted to validate the proposed model and assess its fit.

### 2.3 Phase 3: psychometric analysis of the questionnaire

#### 2.3.1 Reliability analysis

The mean score obtained on the scale was 3.04, with a standard deviation of 0.05, indicating an adequate dispersion of responses. The internal consistency of the instrument was assessed using Cronbach’s alpha and McDonald’s omega coefficients, both yielding a value of 0.96. These results indicate excellent internal reliability and high measurement stability. In addition, the confidence intervals were narrow, which reinforces the precision of the estimates (see Table 1). According to the criteria proposed by Nunnally (1978), these values clearly exceed the recommended threshold of 0.80 for acceptable reliability.

**Table 1:** Frequentist scale reliability statistics.

			**95% CI**	
**Coefficient**	**Estimate**	**Std. Error**	**Lower**	**Upper**
Coefficient ω	0.96	2.37e-3	0.96	0.97
Coefficient *α*	0.96	2.77e-3	0.95	0.96
Mean	3.04	0.05	2.95	3.14

#### 2.3.2 Exploratory factor analysis

Before conducting the analysis of the questionnaire’s internal structure, sampling adequacy was assessed using the Kaiser-Meyer-Olkin (KMO) index, which yielded an excellent value of 0.98. In addition, Bartlett’s test of sphericity was significant (*χ^2^* = 8190.03; *df* = 153; *p* < 0.001), confirming the suitability of the data for factor analysis. Based on this, an exploratory factor analysis (EFA) (Browne, 2001; Ferrando and Lorenzo-Seva, 2018) was conducted using the principal axis factoring method with promax rotation.

The analysis revealed the presence of two factors with eigenvalues greater than 1, which together explained 61% of the total variance (Factor 1 = 50%, Factor 2 = 11%). The factor loadings were adequate, with saturations above 0.60 for most items. The first factor, identified as Internal Logic, grouped items such as P1, P3, P4, P13, P16, P26, P27, and P36, with loadings ranging from 0.70 to 1.01. The second factor, External Logic, included items P5, P6, P18, and P28, with more moderate, yet consistent, loadings within this component (Table 2).

**Table 2:** Factor loadings.

Items	Factor 1	Factor 2	Uniqueness
I L 14	1.01		0.19
I L 8	0.99		0.19
I L 10	0.91		0.20
I_L_2	0.80		0.35
I_L_12	0.79		0.23
I L 11	0.78		0.23
I L 1	0.72		0.32
I L 3	0.71		0.38
I L 9	0.70		0.36
I_L_13	0.70		0.36
I_L_6	0.70		0.37
I L 4	0.63		0.41
I L 7	0.61		0.39
I L 5	0.57		0.38
E_L_4		0.65	0.65
E_L_1		0.54	0.72
E L 2		0.52	0.68
E L 3		0.43	0.64

The structure matrix confirmed a high correlation between the two factors (*r* = 0.77). The model fit indices for the factorial structure were satisfactory: RMSEA = 0.05 (90% CI [0.043, 0.058]), SRMR = 0.02, TLI = 0.97, and CFI = 0.98, indicating a good model fit (Table 3).

**Table 3:** Additional fit indices.

RMSEA	RMSEA 90% confidence	SRMR	TLI	CFI	BIC
0.05	0.043–0.058	0.02	0.97	0.98	−463.17

#### 2.3.3 Confirmatory factor analysis

A Confirmatory Factor Analysis (CFA) (Alcaraz-Muñoz et al., 2022; Bandalos, 2021) was conducted using the Diagonally Weighted Least Squares (DWLS) estimator. The bidimensional model, composed of Internal Logic (14 items) and External Logic (4 items), demonstrated an adequate fit.

The chi-square statistic was significant (*χ^2^* (134) = 521.28, *p* < 0.001), but the fit indices were satisfactory: RMSEA = 0.05, SRMR = 0.02, TLI = 0.97, CFI = 0.98.

The unstandardized factor loadings ranged from 0.92 to 1.24, all of which were statistically significant (*p* < 0.001). The latent variances were also significant: Internal Logic = 0.58 (*p* < 0.001), External Logic = 0.33 (*p* < 0.001). The covariance between the two factors was 0.40 (*p* < 0.001).

**Figure 1:**
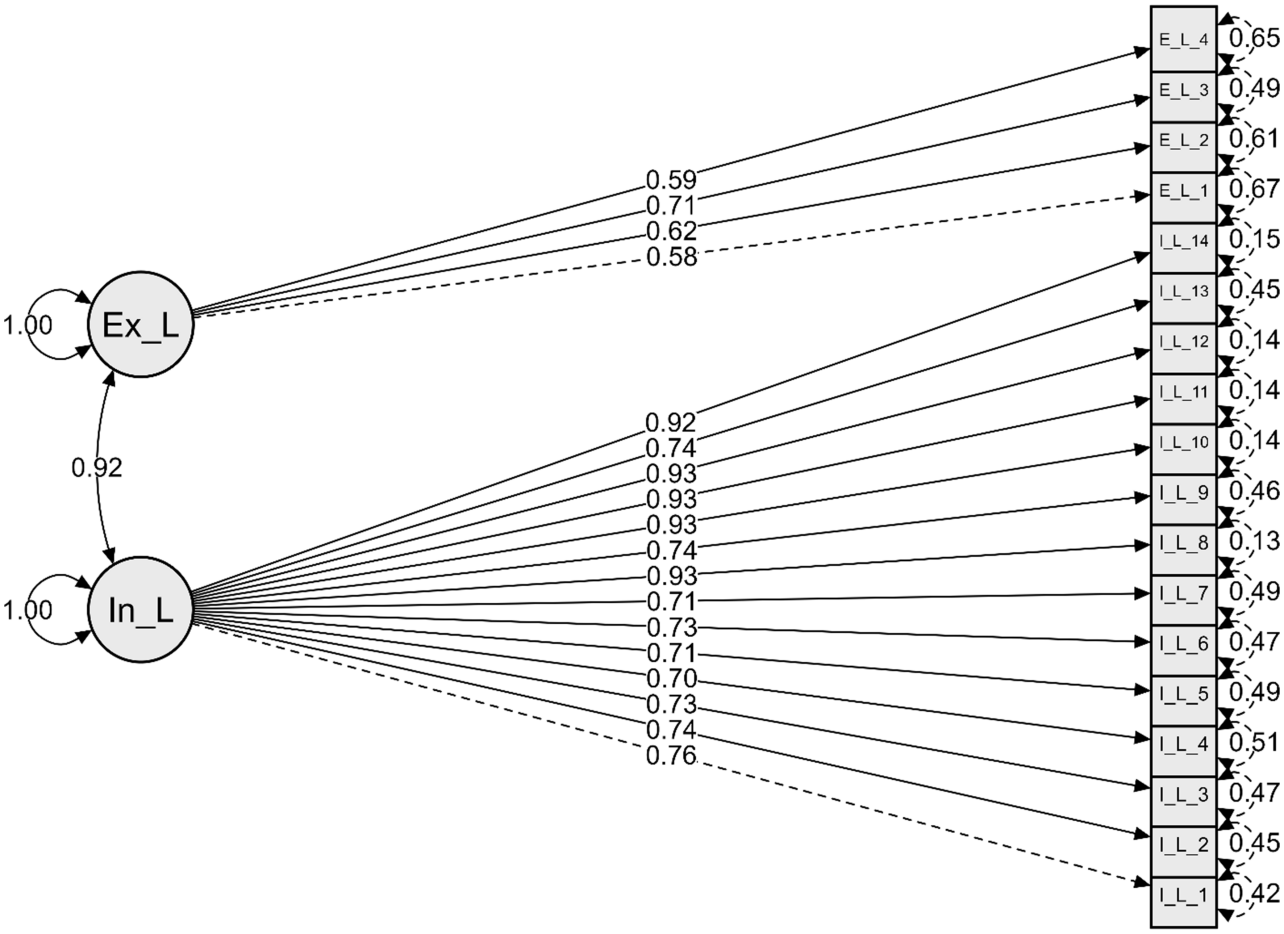
Confirmatory factor model with two factors.

In the diagram (Figure 1), the number on each arrow (e.g., 0.76, 0.73, 0.59) corresponds to the standardized factor loading. This value indicates the strength of the relationship between the factor and the item. Values closer to 1 (or −1) suggest that the item is a strong indicator of that factor. Values closer to 0 indicate that the item does not adequately measure the factor. The arrow from L_I to P1 has a value of 0.76, meaning that item P1 has a strong and positive relationship with the Internal Logic factor. Item P13 has a loading of 0.93, making it an even stronger indicator of Internal Logic. Figure 1 presents our two-factor model. Rectangles such as P1 or P28 represent items, within their respective Internal Logic or External Logic factors (circles). The arrows from circles to rectangles show item loadings; for example, P13 is an excellent indicator of L_I with a loading of 0.93. The numbers to the right, such as 0.13 in P13, indicate measurement error, which in this case is very low. Additionally, a high correlation (0.92) between the two factors is also shown.

## 3 Results

The results are presented in four sections: item descriptive analysis, content validity, exploratory factor analysis (EFA), and reliability analysis.

Regarding the descriptive analysis, item means ranged from 3.90 to 4.50, with standard deviations between 0.70 and 0.90. Most items showed skewness and kurtosis values within the acceptable range of −1 to +1, indicating an approximately normal distribution of responses and the absence of significant bias (Table 4).

**Table 4:** Descriptive statistics of the items.

Descriptive statistics				
Items	Mean	Std. deviation	Skewness	Kurtosis
I_L_1	2.99	1.32	−0.44	−1.36
I_L_2	2.98	1.33	−0.41	−1.38
I_L_3	3.03	1.31	−0.49	−1.28
E_L_1	2.58	1.46	0.40	−1.25
E_L_2	2.51	1.43	0.49	−1.11
I_L_4	3.01	1.33	−0.44	−1.37
I_L_5	3.12	1.33	−0.57	−1.18
I_L_6	2.99	1.33	−0.42	−1.37
I_L_7	3.06	1.32	−0.50	−1.24
I_L_8	3.44	1.71	−0.39	−1.60
I_L_9	3.05	1.33	−0.50	−1.27
I_L_10	3.43	1.71	−0.38	−1.62
E_L_3	2.53	1.49	0.41	−1.31
I_L_11	3.51	1.67	−0.45	−1.53
I_L_12	3.51	1.69	−0.47	−1.54
E_L_4	2.58	1.43	0.37	−1.24
I_L_13	3.02	1.32	−0.47	−1.30
I_L_14	3.45	1.71	−0.38	−1.62

Content validity was assessed through expert judgment (*n* = 13), in which each item was evaluated for clarity, relevance, and alignment with the questionnaire’s objectives. All items obtained a Content Validity Index (CVI) above 0.80, indicating high representativeness and consistency with the evaluated construct (Table 5).

**Table 5:** Content validity index (CVI) by expert panel.

Expert Panel	Clarity	Relevance	Adequacy	Total CVI
Panel 1	0.85	0.90	0.86	0.87
Panel 2	0.88	0.91	0.88	0.89

The combined results from the Exploratory Factor Analysis (EFA) and Confirmatory Factor Analysis (CFA) supported a robust bifactorial structure of the questionnaire. The EFA revealed two well-defined factors with consistent factor loadings, while the CFA confirmed this structure with satisfactory fit indices. The factor variances estimated in the CFA were statistically significant, with narrow confidence intervals (see Table 6), reinforcing the internal stability and coherence of the proposed model.

**Table 6:** Factor variances with 95% confidence intervals.

Factor	Estimate	Standard error	*z*	*p*	Lower CI	Upper CI
Internal Logic	0.58	0.02	23.87	0.000	0.53	0.63
External Logic	0.33	0.04	8.75	0.000	0.26	0.41

The instrument demonstrated excellent internal reliability. As detailed in Table 1, both Cronbach’s alpha and McDonald’s omega coefficients reached a value of 0.96. These results reinforce the psychometric strength of the questionnaire, evidencing its high internal consistency and precision in assessing the proposed construct.

## 4 Discussion

The aim of this study was to develop and validate the CHEAT-1 questionnaire, an instrument designed to assess students’ perceptions of cheating in traditional sporting games (TSG). The findings support the validity and reliability of the instrument, confirming its relevance as an assessment tool.

A suitable, coherent, and adjusted validation analysis procedure has been proposed for a specific type of data; framed within movement sciences. For this reason, the criteria of internal logic and external logic of the items are of paramount importance. There is a need for rigorous studies in the design and validation of questionnaires using traditional games (Moya-Higueras et al., 2025).

The results from both exploratory and confirmatory factor analyses confirmed a well-defined two-dimensional structure (Raykov and Marcoulides, 2011), consistent with the theoretical distinction between Internal Logic and External Logic of TSG. The absence of cross-loadings and the strength of factor loadings support the internal coherence of the scale. Additionally, the explained variance and statistically significant factor variances reinforce the robustness of the underlying construct (Browne, 2001).

Content validity was established through a rigorous multi-phase process, including expert review and student focus groups, ensuring alignment between the items and the theoretical framework of motor conduct and rule transgression. Descriptive statistics indicated an appropriate distribution of responses, with no critical skewness or kurtosis, thus supporting the quality of the dataset.

Internal consistency indices—Cronbach’s alpha and McDonald’s omega—both reached values of 0.96, well above the accepted thresholds for reliability in social sciences. These findings align with previous research on ethical behavior in sport (Alcaraz-Muñoz et al., 2022) and confirm the instrument’s capacity to measure perceptions of cheating with precision and stability.

The final version of CHEAT-1, composed of 18 items across two dimensions, offers a concise yet comprehensive tool that can be applied across educational stages. Its design allows educators and researchers to identify attitudes and beliefs related to fair play, providing insights that can inform pedagogical interventions aimed at promoting ethical engagement in motor games (Table 7).

**Table 7:** CHEAT-1 final version (in Spanish and English translation in italics).

**Item**	**Statement**
I_L_1	Hago trampas al ayudar a mi equipo mientras jugamos. (*I cheat by helping my team while we play*.)
I_L_2	Hago trampas para tener éxito o ganar en el juego. (*I cheat to succeed or win the game*.)
I_L_3	Hago trampas para defender o proteger el material que necesito en el juego (*I cheat to defend or protect the equipment I need in the game*.).
I_L_4	Hago trampas al jugar contra un rival. (*I cheat when playing against an opponent*.)
I_L_5	Hago trampas para no fracasar o perder en el juego. (*I cheat so as not to fail or lose the game*.)
I_L_6	Hago trampas para conseguir el material que necesito en el juego. (*I cheat to get the equipment I need in the game*.)
I_L_7	Hago trampas cuando no puedo defender o proteger un espacio de juego. (*I cheat when I cannot defend or protect a playing space*.)
I_L_8	Hago trampas para tener ventaja en el juego. (*I cheat to gain an advantage in the game*.)
I_L_9	Hago trampas cuando intento conseguir un espacio de juego. (*I cheat when trying to gain a playing space*.)
I_L_10	Hago trampas para que el rival no tenga ventaja en el juego. (*I cheat so that my opponent does not have an advantage in the game*.)
I_L_11	Respeto las reglas al ayudar a mi equipo mientras jugamos. (*I respect the rules when helping my team while we play*.)
I_L_12	Respeto las reglas aunque no tenga éxito ni gane en el juego. (*I respect the rules even if I do not succeed or win in the game*.)
I_L_13	Respeto las reglas aunque fracase o pierda en el juego. (*I respect the rules even if I fail or lose in the game*.).
I_L_14	Respeto las reglas aunque no tenga ventaja en el juego. (*I respect the rules even if I do not have an advantage in the game*.)
E_L_1	Hago trampas para no sentirme apartado del juego. (*I cheat so I do not feel left out of the game*.)
E_L_2	Hago trampas si me permite sobresalir sobre los demás en el juego. (*I cheat if it allows me to stand out from others in the game*.)
E_L_3	Hago trampas para demostrar que soy mejor que los demás en el juego. (*I cheat to prove that I am better than others in the game*.)
E_L_4	Hago trampas porque me presionan para hacerlas. (*I cheat because I am pressured to do so*.)

Despite its strengths, the study presents certain limitations. The use of non-probabilistic sampling restricts the generalizability of results, and no test–retest reliability analysis was conducted to evaluate temporal stability. Future research should address these gaps and explore the instrument’s performance in culturally diverse contexts. Although the factors of internal logic and external logic are highly rigorous supraconcepts of motor action, they are nonetheless very broad macroconcepts. The existence of other Parlebasian notions could make it possible to explore conflicts in traditional games through other items and/or, where appropriate, to construct factors. Different dimensions of motor action, or the network of motor communication among other Universals (Parlebas, 2020), could represent opportunities to build questionnaire validation from other perspectives, while still taking motor conflict as the pedagogical epicenter.

In summary, CHEAT-1 constitutes a psychometrically sound, theory-driven instrument that responds to a significant gap in the literature on ethical education through Physical Education. Its application can contribute meaningfully to understanding and addressing cheating behaviors in motor contexts, fostering the development of fairer, more respectful learning environments.

## Data Availability

The raw data supporting the conclusions of this article will be made available by the authors, without undue reservation.

## References

[ref1] Alcaraz-Muñoz V. Alonso J. I. Yuste J. L. (2022). Design and validation of games and emotions scale for children (GES-C). Cuad. Psicol. Deporte 22, 28–43. doi: 10.6018/cpd.476271

[ref2] American Educational Research Association, American Psychological Association, and National Council on Measurement in Education (2014). Standards for educational and psychological testing. Washington: American Educational Research Association.

[ref3] Bandalos D. L. (2021). Item meaning and order as causes of correlated residuals in confirmatory factor analysis. Struct. Equ. Model. 28, 1–11. doi: 10.1080/10705511.2021.1916395

[ref4] Bermejo J. M. Borrás P. A. Ponseti F. J. (2019). El fair play en edad escolar: Programa “Ponemos valores al deporte”. Rev. Iberoam. Cienc. Act. Fís. Deporte 8, 59–67. doi: 10.24310/riccafd.2019.v8i1.6863

[ref5] Boardley I. D. Kavussanu M. (2007). Development and validation of the moral disengagement in sport scale. J. Sport Exerc. Psychol. 29, 608–628. doi: 10.1123/jsep.29.5.608, PMID: 18089895

[ref6] Boateng G. O. Neilands T. B. Frongillo E. A. Melgar-Quiñonez H. R. Young S. L. (2018). Best practices for developing and validating scales for health, social, and behavioral research: a primer. Front. Public Health 6, 1–18. doi: 10.3389/fpubh.2018.00149 29942800 PMC6004510

[ref7] Browne M. W. (2001). An overview of analytic rotation in exploratory factor analysis. Multivar. Behav. Res. 36, 111–150. doi: 10.1207/S15327906MBR3601_05

[ref8] Bücker S. Nuraydin S. Simonsmeier B. A. Schneider M. Luhmann M. (2018). Subjective well-being and academic achievement: a meta-analysis. J. Res. Pers. 74, 83–94. doi: 10.1016/j.jrp.2018.02.007

[ref9] Bukowski W. M. Brendgen M. Vitaro F. (2007). “Peers and socialization: effects on externalizing and internalizing problems” in Handbook of socialization: Theory and research. eds. Grusec J. E. Hastings P. D. (New York: Guilford Press), 355–381.

[ref10] Cejudo J. Losada L. Feltrero R. (2020). Promoting social and emotional learning and subjective well-being: impact of the “Aislados” intervention program in adolescents. Int. J. Environ. Res. Public Health 17:609. doi: 10.3390/ijerph17020609, PMID: 31963598 PMC7013551

[ref11] Cronbach L. J. (1951). Coefficient alpha and the internal structure of tests. Psychometrika 16, 297–334. doi: 10.1007/BF02310555

[ref12] de Sáez Ocáriz U. Lavega P. (2013). Transformar conflictos en educación física en primaria a través del juego: Aplicación del índice de conflictividad. Cult. Educ. 25, 549–560. doi: 10.1174/113564013808906928

[ref13] Deci E. L. Ryan R. M. (1985). Intrinsic motivation and self-determination in human behavior. Boston, MA: Springer.

[ref14] Deci E. L. Ryan R. M. (2002). Handbook of self-determination research: University of Rochester Press.

[ref15] Del Rey R. Ortega R. Feria I. (2009). Convivencia escolar: Fortaleza de la comunidad educativa y protección ante la conflictividad escolar. Rev. Interuniv. Form. Profesor. 23, 159–180. http://hdl.handle.net/11441/16928

[ref16] Delors J. (1996). Learning: The treasure within. Report to UNESCO of the international commission on education for the twenty-first century: UNESCO.

[ref17] Domitrovich C. E. Durlak J. A. Staley K. C. Weissberg R. P. (2017). Social-emotional competence: an essential factor for promoting positive adjustment and reducing risk in school children. Child Dev. 88, 408–416. doi: 10.1111/cdev.12739, PMID: 28213889

[ref18] Durlak J. A. Weissberg R. P. Dymnicki A. B. Taylor R. D. Schellinger K. (2011). The impact of enhancing students’ social and emotional learning: a meta-analysis of school-based universal interventions. Child Dev. 82, 405–432. doi: 10.1111/j.1467-8624.2010.01564.x, PMID: 21291449

[ref19] Dweck C. S. Leggett E. L. (1988). A social-cognitive approach to motivation and personality. Psychol. Rev. 95, 256–273. doi: 10.1037/0033-295X.95.2.256

[ref20] Eisman A. B. Zimmerman M. A. Kruger D. Reischl T. M. Miller A. L. Franzen S. P.. (2016). Psychological empowerment among urban youth: measurement model and associations with youth outcomes. Am. J. Community Psychol. 58, 410–421. doi: 10.1002/ajcp.12091 27709632 PMC5161682

[ref21] Elliot A. J. Dweck C. S. (Eds.) (2005). Handbook of competence and motivation. New York: Guilford Press.

[ref22] Ferrando P. J. Lorenzo-Seva U. (2018). Assessing the quality and appropriateness of factor solutions and factor score estimates in exploratory item factor analysis. Educ. Psychol. Meas. 78, 762–780. doi: 10.1177/0013164417719308, PMID: 32655169 PMC7328234

[ref23] Festinger L. (1954). A theory of social comparison processes. Hum. Relat. 7, 117–140. doi: 10.1177/001872675400700202

[ref24] Frías-Armenta M. Rodríguez-Macías J. C. Corral-Verdugo V. Caso-Niebla J. García-Arizmendi V. (2018). Restorative justice: a model of school violence prevention. Sci. J. Educ. 6, 39–45. doi: 10.11648/j.sjedu.20180603.11

[ref25] Gibbons S. L. Ebbeck V. Weiss M. R. (1995). Fair play for kids: effects on the moral development of children in physical education. Res. Q. Exerc. Sport 66, 247–255. doi: 10.1080/02701367.1995.10608839, PMID: 7481086

[ref26] Greenberg M. T. Domitrovich C. E. Weissberg R. P. Durlak J. A. (2017). Social and emotional learning as a public health approach to education. Futur. Child. 27, 13–32. doi: 10.1353/foc.2017.0001

[ref27] Gutiérrez-Marín E. Gil-Madrona P. Prieto-Ayuso A. Díaz-Suárez A. (2017). Conductas apropiadas en Educación Física y el deporte en la escuela y validación de la escala. Cuad. Psicol. Deporte 17, 99–110. Available at: https://revistas.um.es/cpd/article/view/301951/216631

[ref28] Herrera M. Romera E. M. Ortega R. Gómez O. (2016). Influence of social motivation, self-perception of social efficacy and normative adjustment in the peer setting. Psicothema 28, 32–39. doi: 10.7334/psicothema2015.167 26820421

[ref29] Hodge K. Lonsdale C. (2011). Prosocial and antisocial behavior in sport: the role of coaching style, autonomous vs. controlled motivation, and moral disengagement. J. Sport Exerc. Psychol. 33, 527–547. doi: 10.1123/jsep.33.4.527, PMID: 21808078

[ref30] Hromek R. Roffey S. (2009). Promoting social and emotional learning with games: “it’s fun and we learn things.”. Simul. Gaming 40, 626–644. doi: 10.1177/1046878109333793

[ref31] Kavussanu M. (2006). Motivational predictors of prosocial and antisocial behaviour in football. J. Sports Sci. 24, 575–588. doi: 10.1080/02640410500190825, PMID: 16611569

[ref32] Kavussanu M. Boardley I. D. (2009). The prosocial and antisocial behavior in sport scale. J. Sport Exerc. Psychol. 31, 97–117. doi: 10.1123/jsep.31.1.97, PMID: 19325190

[ref33] Kirk D. (2020). Precarity, critical pedagogy and physical education. London, UK: Routledge.

[ref34] Lagardera F. Lavega P. (2005). La educación física como pedagogía de las conductas motrices. Tándem. Didáctica Educ. Física 18, 79–102.

[ref35] Latorre-Román P. Á. Bueno-Cruz M. T. Martínez-Redondo M. Salas-Sánchez J. (2020). Prosocial and antisocial behaviour in school sports. Apunts. Educ. Física Deportes 139, 10–19. doi: 10.5672/apunts.2014-0983.es.(2020/1).139.02

[ref36] Lederach J. P. (1995). Preparing for peace: Conflict transformation across cultures. New York: Syracuse University Press.

[ref37] López-Castedo A. Álvarez-García D. Domínguez-Alonso J. Álvarez Roales E. (2018). Expressions of school violence in adolescence. Psicothema 30, 395–400. doi: 10.7334/psicothema2018.86 30353840

[ref38] Martinek T. Lee O. (2012). From community gyms to classrooms: a framework for values-transfer in schools. J. Phys. Educ. Recreat. Dance 83, 33–51. doi: 10.1080/07303084.2012.10598709

[ref39] Martínez Sánchez I. Goig Martínez R. González González D. Álvarez Rodríguez J. (2019). School bullying in compulsory and advanced secondary education: determining factors in its intervention. Int. J. Environ. Res. Public Health 16:750. doi: 10.3390/ijerph16050750, PMID: 30832277 PMC6427412

[ref40] Menéndez Santurio J. I. Fernández-Río J. (2016). Violence, responsibility, friendship and basic psychological needs: effects of a sport education and teaching for personal and social responsibility program. Rev. Psicol. 21, 245–260. doi: 10.1387/RevPsicodidact.14808

[ref41] Moya-Higueras J. March-Llanes J. Sala-Galindo A. Rius J. Estrada-Plana V. Badia-Bafalluy A.. (2025). “Negative attitudes towards gender in traditional sporting games (NATGEN-TSG) inventory” in Promoting sustainable development goals in physical education: The role of motor games. eds. Lavega-Burgués P. Pic M. (Pennsylvania, EEUU: IGI Global Scientific Publishing), 357–384. doi: 10.4018/979-8-3693-6084-2.ch015

[ref42] Norwalk K. E. Hamm J. V. Farmer T. W. Barnes K. L. (2016). Improving the school context of early adolescence through teacher attunement to victimization. J. Early Adolesc. 36, 989–1009. doi: 10.1177/0272431615594466 28042195 PMC5199020

[ref43] Nuñez J. L. Martín-Albo J. Navarro J. G. González V. M. (2006). Validación de la versión española de la Escala de Orientación al Juego Limpio en Educación Física. Rev. Psicol. Deporte 15, 9–23.

[ref44] Nunnally J. C. (1978). “An overview of psychological measurement” in Clinical diagnosis of mental disorders: A handbook, 97–146.

[ref45] Oberle E. Domitrovich C. E. Meyers D. C. Weissberg R. P. (2016). Establishing systemic social and emotional learning approaches in schools: a framework for schoolwide implementation. Camb. J. Educ. 46, 277–297. doi: 10.1080/0305764X.2015.1125450

[ref46] Panayiotou M. Humphrey N. Wigelsworth M. (2019). An empirical basis for linking social and emotional learning to academic performance. Contemp. Educ. Psychol. 56, 193–204. doi: 10.1016/j.cedpsych.2019.01.006

[ref47] Parlebas (2001). Juegos, deporte y sociedad. Léxico de praxiología motriz. Barcelona: Paidotribo.

[ref48] Parlebas P. (2020). The universals of games and sports. Front. Psychol. 11:593877. doi: 10.3389/fpsyg.2020.593877, PMID: 33192937 PMC7609522

[ref49] Raykov T. Marcoulides G. A. (2011). Introduction to psychometric theory. New York: Routledge.

[ref50] Ryan R. M. Deci E. L. (2000). Self-determination theory and the facilitation of intrinsic motivation, social development, and well-being. Am. Psychol. 55, 68–78. doi: 10.1037/0003-066X.55.1.68, PMID: 11392867

[ref9001] Sáez de Ocáriz U. (2011). Conflictos y educación física a la luz de la praxiología motriz. Estudio de caso de un centro educativo de primaria [Tesis doctoral, Universidad de Lleida]. Available at: https://www.tdx.cat/handle/10803/53637#page=1

[ref51] Sáez de Ocáriz U. Lavega P. March J. (2013). El profesorado ante los conflictos en la educación física: El caso de los juegos de oposición en primaria. Revista Electrón. Int. Formación Profes. 16, 163–176. doi: 10.6018/reifop.16.1.183401

[ref52] Shields D. L. L. Gardner D. E. Bredemeier B. J. L. Bostrom A. (1995). Leadership, cohesion, and team norms regarding cheating and aggression. Sociol. Sport J. 12, 324–336. doi: 10.1123/ssj.12.3.324

[ref53] Suls J. Martin R. Wheeler L. (2002). Social comparison: why, with whom, and with what effect? Curr. Dir. Psychol. Sci. 11, 159–163. doi: 10.1111/1467-8721.00191

[ref54] Tajfel H. (1982). Social psychology of intergroup relations. Annu. Rev. Psychol. 33, 1–39. doi: 10.1146/annurev.ps.33.020182.000245

[ref55] Tajfel H. Turner J. C. (1979). An integrative theory of intergroup conflict. Austin W. G. Worchel S. (Eds.), The social psychology of intergroup relations (33–37). Monterey, CA: Brooks/Cole.

[ref56] Teraoka E. Jancer Ferreira H. Kirk D. Bardid F. (2021). Affective learning in physical education: a systematic review. J. Teach. Phys. Educ. 40, 460–473. doi: 10.1123/jtpe.2019-0164

[ref57] Trigueros R. Cangas A. J. Aguilar-Parra J. M. Álvarez J. F. García-Más A. (2019). No more bricks in the wall: adopting healthy lifestyles through physical education classes. Int. J. Environ. Res. Public Health 16:4860. doi: 10.3390/ijerph16244860 31816835 PMC6926670

[ref58] UNESCO (2013). “Declaración de Berlín” in MINEPS V (Paris, Francia: UNESCO).

[ref59] UNESCO. (2014). 37 C/4 Estrategia a Plazo Medio (2014–2021). UNESCO.

[ref60] UNESCO (2015). Educación física de calidad (EFC): Guía para los responsables políticos. Paris, Francia: UNESCO.

